# Encapsulation of Volatile Monoterpene Fragrances in Mesoporous Organosilica Nanoparticles and Potential Application in Fruit Preservation

**DOI:** 10.3390/nano13010104

**Published:** 2022-12-25

**Authors:** Yuanjiang Zhao, Tianwen Bai, Yuhang Liu, Yichao Lv, Zhuxian Zhou, Youqing Shen, Liming Jiang

**Affiliations:** 1 MOE Key Laboratory of Macromolecular Synthesis and Functionalization, Department of Polymer Science and Engineering, Zhejiang University, Hangzhou 310027, China; 2 Key Laboratory of Biomass Chemical Engineering of Ministry of Education and Zhejiang Key Laboratory of Smart Biomaterials, College of Chemical and Biological Engineering, Zhejiang University, Hangzhou 310027, China

**Keywords:** periodic mesoporous organosilicas, nanoencapsulation, nanocomposite, *D*-Limonene, sustained release, fruits and vegetables preservation

## Abstract

In this work, we synthesized mesoporous silica nanoparticles (MSNs) and periodic mesoporous organosilica nanoparticles containing bridging groups of ethylene (E-PMO) and phenylene (P-PMO) and compared their adsorption properties using *D*-limonene (Lim), myrcene (Myr), and cymene (Cym) as model guest molecules. For the selected nanoparticles of ~100 nm in diameter, the loading capacity to the volatile fragrances was in the order of P-PMO < E-PMO < MSN, consistent with the trend of increasing total pore volume. For example, P-PMO, E-PMO, and MSN had a Lim uptake of 42.2 wt%, 47.3 wt%, and 62.7 wt%, respectively, which was close to their theoretical adsorption capacity. Under isothermal thermogravimetric analysis conditions (30 °C, a N_2_ flow of 1 mL min^−1^), the lowest fragrance release of ~56% over 24 h was observed for P-PMO, followed by E-PMO (74–80%), and MSN (~89%). The release kinetics of the fragrant molecules from MSN and PMO materials can be well described by first-order and Weibull models, respectively. Moreover, the incorporation of Lim-loaded P-PMO NPs in an aqueous solution of regenerated silk fibroin provided a composite coating material suitable for perishable fruit preservation. The active layer deposited on fruit peels using dip coating showed good preservation efficacy, enabling the shelf-life of mangoes in a highly humid and hot atmosphere (30–35 °C, 75–85% RH) to be extended to 6 days.

## 1. Introduction

*D*-limonene is the major constituent in citrus essential oils, which has a pleasant and lemon-like smell. It is used as a common flavoring additive in cosmetics, foods, and pharmaceuticals because of its fragrance and proved safety for humans [1,2,3]. The compound has also been considered as a sustainable alternative to synthetic preservatives and pesticides due to its antioxidant, antimicrobial, and insecticidal activities when used alone or in combination with other substances [4,5,6,7,8,9]. Nevertheless, incorporation or encapsulation of *D*-limonene into an appropriate material matrix is a fundamental step for the practical implementation of its various applications, which would facilitate a reduction in evaporation losses and oxidative degradation during storage [10,11,12,13].

Over the last decade, the use of nanotechnology in the protection of flavors and fragrances has drawn considerable research interest owing to the unique properties of nanocarriers [12,14,15,16,17]. By decreasing the size of the carrier from micrometers to nanometers, the surface-to-volume ratio can be significantly increased, leading to higher encapsulation efficiency, enhanced adhesion, and controlled release of entrapped active ingredients [16,18]. The common material matrices employed for nanoencapsulation of limonene are those of sustainable polymers such as chitosan [19], amylose [20], zein [21], and some protein–polysaccharide complexes [15,22,23]. Moreover, inorganic particles, e.g., mesoporous silica nanoparticles (MSNs) [24,25,26], zeolites [27], or organic–inorganic hybrid materials such as metal–organic frameworks [28,29,30,31,32,33,34], have been shown to enclose volatile fragrances or essential oils efficiently and slow the evaporation of cargos confined within the nanopores. These inorganic or hybrid nanomaterials have innate advantages over polymeric nanocapsules, including structural rigidity, high loading capacity, and, in many instances, compatibility with aromatic species [11,30]. However, there is still a limited number of research studies on the entrapment of highly volatile terpene-type fragrance oils such as limonene using porous nanomaterials [14]. In addition, the existing limonene delivery systems based on organic and inorganic nanocarriers usually have a relatively low loading capacity [10,14,26,28,29], which may not offer an effective concentration or enough action duration in some application scenarios [4].

Periodic mesoporous organosilica nanoparticles (PMO NPs) are more recently developed structural analogues of MSNs, distinguished by the presence of bridging organic moieties within the silica framework [35]. In addition to the typical porous structural features of mesoporous materials, such as large surface area, high pore volume, and tunable pore size, PMOs feature molecularly organic–inorganic hybrid compositions where versatile organic moieties are directly incorporated into the framework via covalent linkages, i.e., an organic-bridged silsesquioxane framework, making them much more attractive and useful in comparison to purely inorganic mesoporous silica materials with a siloxane network [36,37].

**Scheme 1:**
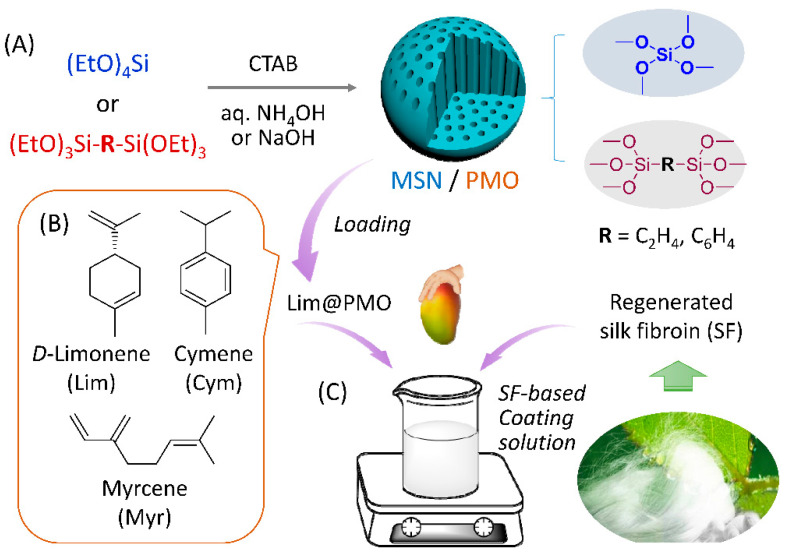
(**A**) Schematic representation for the synthetic procedure of mesoporous silica (MSN) and periodic mesoporous organosilica (PMO) nanoparticles and their structures. (**B**) The molecular structure of the model fragrances studied in the present work. (**C**) An SF-based nanocomposite coating solution prepared by mixing Lim@PMO with regenerated silk fibroin (SF) in water for the preservation of mangoes.

With the objective to explore the capability and features of PMO materials as a nanocarrier for volatile scented organics, herein we synthesized silica-based porous materials with a different constitution, namely, MSNs and ethylene- and phenylene-bridged PMO NPs, and examined their properties to adsorb and retain the model fragrance oils *D*-limonene, myrcene, and cymene (Scheme 1). The rationale for the choice of the three bioactive compounds rested on the possibility of probing the relationship between the aroma retention and mesopore structure of the matrices. *D*-limonene (Lim) and myrcene (Myr) are monocyclic and acyclic terpenes, respectively, while cymene (Cym) represents an aromatic hydrocarbon; thus, this difference in molecular skeletons is expected to lead to different adsorption and release behaviors, as previously noted with some microporous adsorbents [27,31,33]. After investigating the as-synthesized nanoparticle properties of retention for fragrant liquids, we also preliminarily evaluated the potential utility of Lim-loaded composite nanoparticles in perishable fruit preservation. To be specific, *D*-limonene was pre-encapsulated in PMO NPs followed by dispersion in an aqueous solution of regenerated silk fibroin to produce a nanocomposite coating material. Using simple dip coating, the film formed on the fruit peels was found to be effective in reducing the disease severity and weight loss of the coated mangoes, prolonging the shelf life by up to 6 days in realistic summer environments.

To our knowledge, applications of PMO materials to date have been mostly focused on gas storage and separation, heterogeneous catalysis, enzyme immobilization, and drug delivery [37]. No reports on PMO-based nanodelivery systems have been made for highly volatile bioactive fragrances and food preservation.

## 2. Experiments

### 2.1. Materials and Methods

We purchased 1,2-bis(triethoxysilyl)ethane from Heowns Biochem LLC (Tianjin, China). Tetraethyl orthosilicate (TEOS), cetyltrimethylammonium bromide (CTAB), ethanol, sodium hydroxide, ammonium hydroxide, and hydrochloric acid were supplied by Sinopharm Chemical Reagent Co., Ltd. (Shanghai, China). *D*-limonene, myrcene, and cymene were from Shanghai Aladdin Bio-Chem Technology Co., Ltd. (Shanghai, China) and were purified using distilling prior to use.

NMR spectra were recorded using a Bruker AVANCE III 400 spectrometer (400 MHz for ^1^H NMR and 100 MHz for ^13^C NMR). Chemical shifts are reported in parts per million (ppm) relative to tetramethylsilane (TMS). Fourier transform infrared spectroscopic (FT-IR) spectra were recorded in the range of 4000–500 cm^−1^ using a Nicolet IS10 spectrophotometer (Bruker TENSOR II, Karlsruhe, Germany) with successive scans at a resolution of 2 cm^−1^. Samples were prepared using the KBr pellet method. Dynamic light scattering (DLS) was performed using the ZetaSizer Nano ZS3600 (Malvern Instruments, Malvern, UK) under the scattering angle of 173° at a wavelength of 532 nm to measure the hydrodynamic diameter and zeta potential of the nanoparticles in aqueous media at room temperature. Scanning electron microscope (SEM) were obtained using scanning electron microscope (HITACHI S-4800, HITACHI, Tokyo, Japan). Samples were sputter-coated with gold and imaged at an accelerating voltage of 3 kV. Transmission electron microscopy (TEM) images were taken using an HT7700 transmission electron microscope (Hitachi, Tokyo, Japan) at an acceleration voltage of 100 kV. The samples were suspended in DI water and dropped on a carbon-coated copper grid followed by drying at ambient conditions. The particle size was estimated using the image analysis software ImageJ (ver. 1.51j8; National Institutes of Health, Bethesda, MD, USA) and expressed as a mean ± standard deviation based on 100 random counts. Thermogravimetric analysis (TGA) was conducted using a Q50 thermogravimetric analyzer (TA Instruments, New Castle, DE, USA) at a heating rate of 10 °C min^−1^ from r.t. to 800 °C under an airflow of 0.6 mL min^−1^. Nitrogen adsorption–desorption isotherms were recorded at 77 K using a BELSORP-max automated gas sorption apparatus (MicrotracBEL Corp., Osaka, Japan). Before the measurements, a certain amount of the sample (20–30 mg) was degassed under a vacuum (1 × 10^−5^ Pa) for 12 h at 120 °C. The specific surface area and pore volume were calculated using the Brunauer–Emmett–Teller (BET) equation from the adsorption isotherm branch. The average pore diameter was analyzed using the Barrett–Joyner–Halenda (BJH) model from desorption isotherm data. Powder X-ray diffractometric (PXRD) patterns were measured with an X′Pert^3^ powder diffractometer (PANalytical, Almelo, The Netherlands) using Cu K*α* radiation (*λ* = 1.54 Å), operating at 40 kV, 40 mA in the 2*θ* range of 0.6–10° with a step width of 0.02° and a scan rate of 0.02° min^−1^.

### 2.2. Synthesis of 1,4-Bis(triethoxysilyl)benzene

We synthesized 1,4-di(triethoxysilyl)benzene following the reported methods in [38]. Under a nitrogen atmosphere, a small crystal of iodine was added to a mixture of magnesium turnings (7.5 g, 0.31 mol) and TEOS (225 mL, 1 mol) in THF (150 mL) and the mixture was refluxed for several minutes. To the resulting mixture, a solution of 1,4-dibromobenzene (24 g, 10.2 mmol) in THF (50 mL) was added dropwise over 2 h. After completion of the dibromide addition, the reaction mixture was kept at reflux for 2 h. The grey–green mixture was cooled to room temperature prior to the vacuum removal of the solvent. To extract the desired product, 100 mL of hexane was added, and the mixture was filtered under nitrogen to afford a clear, light brown solution. Hexane and residual TEOS were removed in vacuo to give a brown oil. After fractional distillation (0.2 mmHg, 130–134 °C), the target compound was obtained as a colorless oil (yield 45%). ^1^H NMR (400 MHz, CDCl_3_): *δ* 7.67 (s, 4 H, ArH), 3.86 (q, 12 H, *J* = 7.0 Hz, OCH_2_CH_3_), 1.23 (t, 18 H, *J* = 7.0 Hz, OCH_2_CH_3_); ^13^C NMR (101 MHz, CDCl_3_): *δ* 134.02, 133.16, 58.74, 18.19. The NMR spectra are provided in the Supporting Information (Figure S1).

### 2.3. Synthesis of Mesoporous Silica and Organosilica Nanomaterials

Mesoporous silica nanoparticles (MSNs) were prepared according to the reported procedure in [39]. The experimental details may be found in the Supporting Information.

Mesoporous organosilica (E-PMO and P-PMO) were synthesized using the literature methods in [40] with the minor modifications outlined as follows: E-PMO: CTAB (0.21 g), deionized water (96 mL), and an aqueous solution of ammonium hydroxide (2.6 mL, 27 wt%) were mixed and stirred for 10 min to form a clear solution at room temperature. The resultant solution was heated to 50 °C, to which 1,2-bis(triethoxysilyl)ethane (0.92 mmol) was added dropwise, and then the stirring was continued for 6 h at this temperature. The resulting suspension was cooled to ambient temperature and aged overnight without stirring. The product was isolated by centrifugation (8000 rpm for 10 min; the diameter of the centrifuge rotor = 18.4 cm), washed, and dispersed with deionized water and ethanol several times using ultrasonic. Subsequently, the surfactant-filled particles were solvent-extracted with a hydrochloric acid/ethanol mixture (36 wt% HCl/EtOH 1: 50, *v*/*v*) for 12 h, followed by sonication for 30 min at 50 °C in order to remove the CTAB template, washed twice with ethanol and finally with acetone. The powdered product was additionally dried in a vacuum at 60 °C for 24 h.

P-PMO: CTAB (0.21 g), deionized water (86 mL), *n*-propanol (10 mL) and an aqueous solution of ammonium hydroxide (2 mL, 27 wt%) were mixed and stirred for 10 min to form a clear solution at room temperature. The resultant solution was heated to 80 °C, to which 1,4-bis(triethoxysilyl)benzene (0.76 mmol) was added dropwise, and then the stirring was continued for 2 h at this temperature. The subsequent treatment was performed similarly to that of E-PMO.

### 2.4. Encapsulation of Model Fragrances in NPs

A certain amount of the synthesized particulate material was placed in a flask, degassed at 120 °C for 6 h, and upon cooling to room temperature, filled with dry nitrogen. Then, the fragrance oil was added at a predetermined amount, and the resulting mixture was stirred for 24 h, separated by centrifugation, and rinsed with *n*-hexane several times in a gentle N_2_ flow to remove the oils adsorbed on the solid surface. The obtained composites were transferred into screw-capped glass vials and stored in a desiccator prior to various characterizations. These fragrance-loaded samples are hereinafter referred to as FG@MSM, FG@E-PMO, or FG@P-PMO, where FG represents Lim, Myr, or Cym.

The total amount of fragrance in the resultant nanocomposites was determined using TGA by measuring the weight difference between the pristine carriers and fragrance-loaded NPs. The fragrance loading content (LC, indicated in % by weight) is defined by the following Equation (1):

LC (wt%) = *W*_FG_/*W*_total_ × 100% (1)

where *W*_FG_ is the weight of adsorbed fragrance in carriers, and *W*_total_ represents the total weight of the fragrance-loaded composite.

### 2.5. Fragrance Release Experiments Using Gravimetrical Analysis

The fragrance release was evaluated by monitoring the weight loss of fragrance-loaded composites at regular time intervals using a thermogravimetric analyzer. Briefly, FG-loaded powder samples (50–60 mg) or pure fragrance oils (0.3 mL) were placed in a glass vial, and the gravimetrical change was recorded at a constant temperature (30 ± 0.1 °C) under a nitrogen flow of 1 mL min^−1^. Each cumulative release percentage of fragrance was derived from three independent measurements and was expressed as the mean ± standard deviation.

### 2.6. Computational Methodology

The binding energies of model fragrance molecules to the organic bridging groups of PMOs and to the siloxane bonds in inorganic silica were studied using a quantum chemistry calculation. The geometries of the FG/bridging moiety complexes were optimized using the M06-2X with a 6-31+G(d,p) basis set [41]. Frequency calculations were carried out to ensure all intermediates had zero imaginary frequencies. All calculations were performed using the Gaussian 16 package [42].

### 2.7. Preparation of SF-Based Nanocomposite Coating and Its Preservation Function

Regenerated silk fibroin (SF) was extracted from *Bombyx Mori* cocoon fibers using dissolution in a 9.3 M LiBr solution and dialysis in deionized water [43], the detailed procedure is described in the Supporting Information. A solution of 4.0% (*w*/*v*) SF in deionized water was prepared, followed by dispersing 5% (*w*/*w*) of P-PMO or Lim@P-PMO NPs (*D*-limonene content ~38 wt%) in the solution. The resulting mixture was allowed to stir gently for 30 min to achieve a homogeneous distribution of NPs in the coating formulation. The two colloidal solutions were denoted as P-PMO/SF and Lim@P-PMO/SF, respectively.

Mango fruits (just started to ripen) were collected from local markets and selected to be free of visual defects and uniform in terms of weight/shape (the average weight of each was 100 ± 5 g). A set of five mangoes was coated by dipping in each coating solution (i.e., Lim@P-PMO/SF, SF, and P-PMO/SF) and then allowed to air-dry at r.t. The same process was repeated three times. The coated mangoes along with uncoated samples as a control were kept in ambient conditions (30–35 °C, 75–85% RH), and examined for changes in appearance and weight every day.

## 3. Results and Discussion

### 3.1. Fabrication and Characterization of Nanocarrier Materials

The nanoparticulate materials were synthesized using condensation of silica precursors in basic aqueous media in the presence of mesopore-templating surfactant cetyltrimethylammonium bromide [39,40]. In the case of MSNs, the silica precursor was tetraethoxysilane, while for E-PMOs and P-PMOs, the silica precursors were 1,2-bis(triethoxysilyl)ethane and 1,4-bis(triethoxysilyl)benzene, respectively (Scheme 1A). After washing consecutively with water and ethanol several times, the surfactant was removed using calcination at 550 °C in the case of MSNs or using extraction with acidic ethanol followed by sonication in the case of PMO NPs. The SEM and TEM images showed that all as-prepared materials consisted of nearly spherical particles, with diameters of 82–132 nm, 57–334 nm, and 105–395 nm for MSNs, E-PMO, and P-PMO NPs, respectively (Figures S2–S4 in Supporting Information). The DLS values (Figure S5) were in excellent agreement given that DLS gives the hydrated diameter, while TEM is measured in the nonhydrated state. The particle size of PMO NPs can be adjusted by the variation of the ammonia concentration or the co-solvent content in the reaction mixture, as was the case for those reported earlier [40]. The values of zeta potential for the nanoparticles were between −12.8 ± 0.4 and −24.8 ± 0.2 mV (Tables S1 and S2).

**Figure 1:**
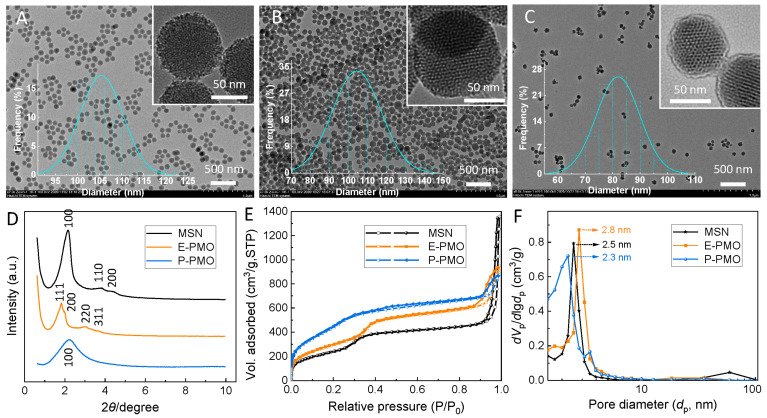
Structure characterization of three representative porous nanomaterials. TEM images of (**A**) P-PMO (*d*_TEM_ = 105.4 ± 4.9 nm), (**B**) E-PMO (*d*_TEM_ = 104.4 ± 14.4 nm), (**C**) MSN (*d*_TEM_ = 81.9 ± 7.9 nm), and histograms of the distribution of particle diameters (based on measurements of ~100 individual particles) as inlays. (**D**) Powder XRD patterns, (**E**) BET nitrogen adsorption–desorption isotherms measured at 77 K and (**F**) the corresponding BJH pore size distribution curves calculated using the BJH method (adsorption branch). Hollow and solid points represent adsorption and desorption data, respectively.

We selected five as-synthesized porous materials with a particle size of 80–120 nm for in-depth characterization in terms of their pore structure, and the relevant data are summarized in Table 1. Figure 1A–C show TEM images of the three samples of ~100 nm diameter (entries 1, 2, and 5 in Table 1) with particle diameter histograms. From the high magnification insets, both MSNs and E-PMO NPs presented a well-ordered hexagonal arrangement of tubular mesopores. However, the pore structure of P-PMO is not as perceptible as the other two materials discussed above, probably because of the nonuniform pore aperture and lower contrast between the organic framework and empty pores in comparison to the siloxane network in MSNs [44].

Figure 1D displays the powder X-ray diffraction (XRD) patterns of the three representative samples. The pattern of MSNs exhibits three well-resolved peaks in the range of 1.5° < 2*θ* < 4.5° that can be indexed as the (100), (110), and (200) reflections, indicative of a long-range ordered hexagonal framework [45]. The XRD pattern of E-PMO NPs presents four diffraction peaks in the 2*θ* angle of 1.5–4°, which correspond to the (111), (200), (220), and (311) reflection planes associated with a face-centered-cubic *Fm*3*m* symmetry [46]. For P-PMO materials, a broad XRD peak assignable to the (100) plane was observed, demonstrating a markedly decreased long-range ordering in comparison to MSN and E-PMO materials [40]. These results are in accord with the above TEM observations.

Nitrogen physisorption revealed a type IV isotherm for the three nanoparticles [47] (Figure 1E). Each sample exhibits an additional capillary condensation of N_2_ at high relative pressures (*P*/*P*_0_ > 0.90), indicating a high degree of textural porosity [48]. Hysteresis loops are hardly observed in the isotherms due to the relatively small pore sizes resulting from the CTAB surfactant [49]. Among these materials, P-PMO has the highest Brunauer–Emmett–Teller (BET) specific surface area (1654 m^2^ g^−1^), followed by E-PMO (1082 m^2^ g^−1^) and MSN (882.5 m^2^ g^−1^). In addition, our results suggested that elevating the reaction temperature from 50 to 80 °C of the sol–gel process with a shortened period of time from 6 to 2 h seems to effectively tune the physisorption data of PMO materials. For instance, P-PMO NPs showed a BET surface area (*S*_BET_) increase of ca. 27% compared to that of materials obtained under 50 °C/6 h at otherwise unchanged conditions (Table 1, entries 5 vs. 4). As for E-PMO materials, the same strategy resulted in a halving of the pore volume and a lower *S*_BET_ value from 1082.3 m^2^ g^−1^ to 904.3 m^2^ g^−1^ (entries 2 vs. 3).

### 3.2. Encapsulation of Fragrances in the Mesoporous Matrices

**Figure 2:**
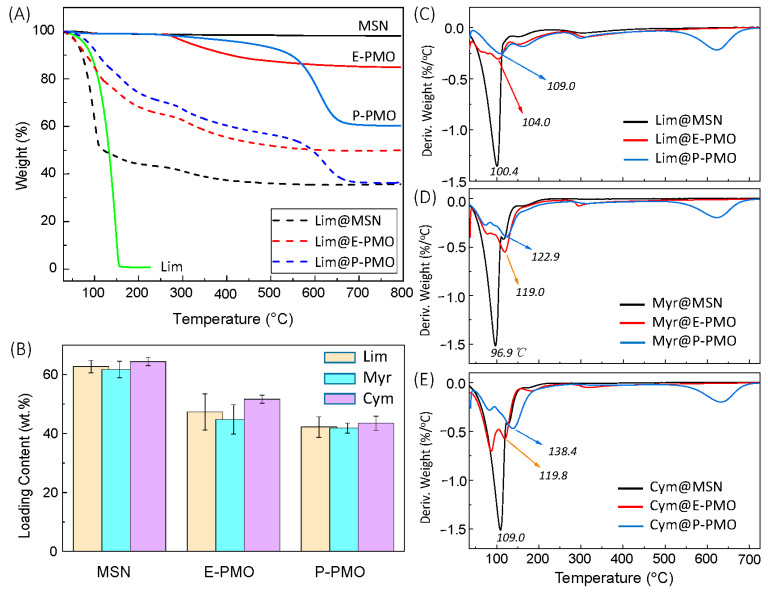
(**A**) Thermal gravity analysis curves of carrier materials, pure *D*-limonene (Lim), and Lim-loaded composites. (**B**) Adsorption capacities of MSN, E-PMO and P-PMO towards model fragrances, determined by TGA. The loading experiments were conducted in the presence of excess fragrance oils. Right panel: derivative thermal gravity analysis (DTG) curves for nanocomposites loaded with (**C**) *D*-limonene, (**D**) myrcene, and (**E**) cymene; where black, blue, and red lines represent MSN, E-PMO, and P-PMO nanoparticles, respectively.

The fragrances were directly incorporated into mesoporous NPs using the impregnation method. The resulting composites were characterized using thermogravimetric analysis (TG) and Fourier transform infrared (FT-IR) spectroscopy to identify the loading of guest molecules in the carriers. Figure 2A shows as an example the TG curves of free *D*-limonene (Lim) and three representative nanoparticles (entries 1, 2, and 5 in Table 1) before and after adsorption of Lim. Overall, these mesoporous materials exhibited a good thermal stability before 250 °C. The weight loss of MSNs was less than 1% in the range of 35–100 °C, ascribed to the desorption of physically adsorbed water. The weight losses of the PMO products started at around 280 °C, probably due to the decomposition of the residual template or a small portion of organic bridging groups [50]. A sharp weight change was observed centered at 274 °C and 611 °C for E-PMO and P-PMO NPs, respectively (see Figure S6), which corresponds to the rapid degradation of hybrid organosilica frameworks. When compared with the pristine carriers, Lim-loaded composites showed about 7–36% more weight loss at 100 °C because of the aroma volatilization. Similar results were observed for the other nanoparticles embedded with Myr and Cym oils (Figure S7). These observations point to the successful encapsulation of fragrances in the porous matrices. This was also evidenced by IR spectra of the composites from the appearance of absorption bands characteristic of aroma compounds (see Figure S8). However, no significant shift was observed for the characteristic infrared absorption of the confined fragrances in comparison with the free fragrances, indicating the absence of strong non-covalent interaction between the guest molecules and the surface of the nanochannels.

The adsorption capacities for fragrances on the MSNs and PMO NPs are plotted in Figure 2B. It could be seen that the loading capacity (LC) to fragrance oils increases in the order P-PMO < E-PMO < MSN, following the opposite trend of the specific surface area (*S*_BET,P-PMO_ > *S*_BET,E-PMO_ > *S*_BET,MSN_) but being proportional to the total pore volume (*V*_p_) of nanoparticles. In fact, the experimental LC values were in good agreement with the predicted values calculated using the formula LC_theor._ = *V*_p_ × ρ_FG_/(1 + *V*_p_ × ρ_FG_) (where ρ_FG_ is the density of fragrance liquid). For example, the three nanoparticles had a Lim uptake of 62.7%, 47.3%, and 42.2%, respectively, being close to the theoretical values 59.8%, 45.1% and 41.3% for MSN, E-PMO, and P-PMO. This means that the pore volume appears to be the main factor in determining the loading capacity of these carriers. With respect to the loading capacity for *D*-limonene, PMO NPs are significantly superior to the previously reported carrier materials such as MSNs [26], zeolites [27], MOFs [28,29] and polymeric nanoparticles [20,21,22,43,51,52].

Figure 2C–E shows the derivative TG (DTG) thermograms of the selected nanocomposites, which provides a clearer insight into the thermal stability using a comparison of the peak temperature (*T*_p_) corresponding to the maximum rate of weight loss. As can be seen, there existed apparent differences among these DTG curves. The *T*_p_ varied with the different types of carriers, and its value increased in the order MSN < E-PMO < P-PMO in all three sets of instances. Remarkably, the observed *T*_p_ shift was consistent with the trend of increasing BET surface area (*S*_BET_) and decreasing average pore diameter (*d*_p_) of the particles (see Table 1). That is, as the pore size is decreased or the surface area is increased, aroma molecules are entrapped more efficiently in the nanostructures. Moreover, for Cym@P-PMO, the observed *T*_p_ of 138.4 °C appeared to be unexpected as compared to the much lower *T*_p_ (109.0 °C) of Lim@P-PMO, considering the fact that the free cymene and *D*-limonene have identical boiling points (176–177 °C) but the saturated vapor pressure of the former is higher than that of the latter (1.7 ± 0.2 mmHg vs. 1.5 ± 0.2 mmHg at 25 °C, respectively [53]). The retarded thermal evaporation of cymene may be partly related to the enhancement of intermolecular interactions due to the π–π stacking of aromatic residues in the constrained spaces.

### 3.3. Kinetics of Fragrance Release

**Figure 3:**
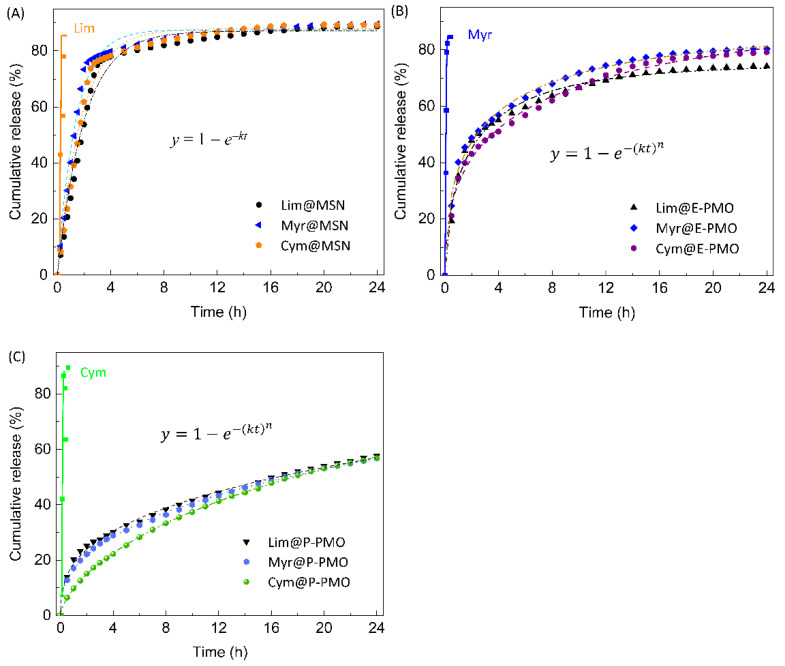
Fragrance release curves (symbol) of nanocomposites based on (**A**) MSN, (**B**) E-PMO NPs, and (**C**) P-PMO NPs, as measured by TGA of the samples at 30 °C under a N_2_ flow of 1.0 mL min^−1^. Dashed lines represent the corresponding fits with the first-order kinetics (**A**) and Weibull models (**B**,**C**), where *y* is the fraction of the released fragrance to the total amount of fragrance at time *t*, *k* is the release rate constant, and *n* is the release exponent. The initial fragrance load of the composites was controlled in the range 36–40 wt%, achieved by soaking excess NPs (1 g) in a limited amount (0.5 mL) of fragrance during the preparation.

The aroma release behavior of the FG-loaded composites was initially studied using isothermal TGA at 30 °C under a N_2_ flow of 1 mL min^−1^. Figure 3 presents the fragrance release profiles obtained under those conditions. On the whole, all of the composites showed a significant increase in the time required to achieve the fragrance release compared to the free fragrance liquids. The 24 h total release fraction of FG@MSN, FG@E-PMO, and FG@P-PMO were ca. 90%, 75–80%, and 58%, respectively, while the neat fragrances (0.3 mL) evaporated fully within 2 h. It is worth noting that PMO-based systems (Figure 3B,C) exhibited a relatively steady and slower release profile, without the burst release phenomenon as observed in the case of FG@MSNs (Figure 3A).

From a view of the release profiles of MSN-based composites (Figure 3A), the data could be well described using a first-order kinetics function (*y* = 1 − e^−*kt*^). In this case, the release rate of entrapped fragrances mainly depends on the initial load and is positively proportional to it [54]. As for the delivery systems based on either E-PMO or P-PMO NPs, the kinetic data were adequately fitted with a Weibull model ($y=1-e^{-{\left(kt\right)}^{n}}$) allowing a release rate constant to be derived from each experiment. Using an adjustment of the experimental data to the mathematical models, the main parameters were obtained for the three systems (Table 2).

The Weibull model is derived from the first-order equation, and it has been applied for the analysis of drug dissolution and release studies [55]. The exponent *n* in the function is a parameter related to the diffusion mechanism of guest molecules. For *n* ≤ 0.75, the diffusion resulted in Fickian diffusion in either fractal (*n <* 0.69) or Euclidian spaces (0.69 *< n <* 0.75), whereas for 0.75 *< n <* 1.0, this followed the combined mechanism (Fickian diffusion and case II transport). As seen from Table 2, the index *n* for the PMO systems is below 0.69, indicating that the aroma evaporation was controlled via a typical Fickian diffusion mechanism in fractal space [56].

Inspection of Table 2 also reveals that among the tested nanocomposites, the best sustained-release performance was achieved with those formed by P-PMO NPs. For instance, the time required to reach a fragrance release at 50% (*t*_50%_) was 16.2 h for Lim@P-PMO (entry 7, Table 2), which is ca. 6.7 and 8.7 times longer than the time observed in the cases of Lim@E-PMO and Lim@MSN (entries 4 and 1, Table 2), respectively. Meanwhile, the rate constant (*k*, h^−1^) changed with the varied carrier, decreasing in the order Lim@MSN (0.4981) > Lim@E-PMO (0.4287) > Lim@P-PMO (0.0203). A lower *k* value reflects a stronger retention capacity, also implying a stronger host–guest interaction [57]. The theoretical calculation showed the same carrier-dependence of the limonene binding energy which were found to be −19.98, −15.04, and −11.99 kcal mol^−1^, respectively, for phenylene-, ethylene-bridged organosilicas, and inorganic siloxane framework (Table 3). The difference in the binding affinity is in agreement with the variation in the release rate constant discussed above, which suggests that the host–guest hydrophobic or π–π stacking interactions played an essential role in aroma retention. Likewise, the other two series of composites containing myrcene and cymene showed similar trends as the Lim-loaded NPs in the release kinetics and the interaction energy (Table 2 and Table 3).

### 3.4. The Preservation Property of Lim@P-PMO/SF Coatings

In view of the high loading capacity and sustained release effect of PMOs on *D*-limonene, it was interesting to assess the potential of Lim@PMO NPs as an active ingredient for antimicrobial packaging materials. To this end, we prepared a composite coating by mixing Lim@P-PMO with an aqueous solution (4.0%, *w*/*v*) of regenerated silk fibroin in a proper ratio. In the formulation, the regenerated silk fibroin (SF) served as a dispersity-enhancer to improve the stability of colloidal dispersions, and the film-forming property inherent to SF fibers will allow the adhesion of nanoparticles onto the peel of fruits. Recently, Omenetto et al. [58] reported the use of a silk fibroin suspension as an edible coating material for the preservation of strawberries and bananas. In the present work, we chose ripe mangoes as an exemplar fruit because they are prone to deterioration [59] and are suitable for direct packaging with coating materials.

**Figure 4:**
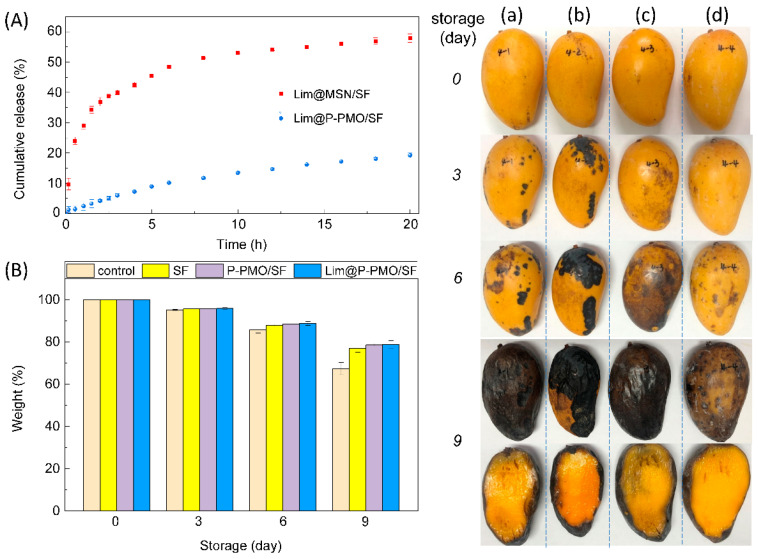
(**A**) Release profiles of SF-coated NPs (■) Lim@MSN/SF and (●) Lim@P-PMO/SF under isothermal TGA conditions (30 °C, a N_2_ flow at 1.0 mL min^−1^). The samples were obtained by dipping Lim@MSN or Lim@P-PMO (LC = 38 ± 1%) in a 4% (*w*/*v*) silk fibroin solution (25 °C, 1 min) followed by freezing and lyophilization. (**B**) Change in weight of mangoes with different coatings and uncoated samples during storage at ambient temperature 30–35 °C and 75–85% RH. Right panel: visual appearance of mango samples during storage, respectively, coated with (**a**) SF, (**c**) P-PMO/SF, (**d**) Lim@P-PMO/SF, and (**b**) without coating as a control. Error bars represent the standard deviation.

We first examined the release profiles of Lim@MSN and Lim@P-PMO nanoparticles coated with regenerated SF layers (Figure 4A). Upon simple dipping followed by freeze drying, the resulting composites offered a slower release compared to their respective bare particles (cf. Figure 3A,C), which can be attributed to the additional contribution of the SF membrane over the particle surfaces. Moreover, SF-coated Lim@P-PMO particles still outperformed their MSN counterparts in terms of aroma retention, in which the cumulative release percentage over 20 h of the former (19.2%) was about one-third of that for the latter (57.9%). We, therefore, took the Lim@P-PMO/SF coating solution as a representative to evaluate its usefulness in the preservation of perishable fruits. The colloidal dispersion was directly deposited on mangoes using dip coating. The initial amount of *D*-limonene adhered to each fruit was ca. 20–25 mg after three dipping-air-dry cycles. After that, the coated mangoes and control samples without coating were stored in a realistic summer environment (30–35 °C, 75–85% relative humidity).

As exemplified in the right panel in Figure 4, the visual quality of the mangoes coated with Lim@P-PMO/SF (d) and P-PMO/SF (c) did not change during the first 3 days of storage at the described conditions, while the SF-coated samples (a), especially ones without coating (b), started to show obvious black spots. The first symptoms of decay (light black spots) occurred for mangoes coated with Lim@P-PMO/SF after day 6. All of the tested fruit samples were spoiled at the end of the 9-day storage period. However, the mangoes coated with Lim@P-PMO/SF had relatively low spoilage as judged from the visible rotten areas of peels and pulps, probably due to the bacteriostatic activity of limonene [7,60,61].

On the other hand, weight loss increased with storage time as expected (Figure 4B). The control presented higher weight loss over time reaching 15% at 6 days of storage, while the coated mangoes showed lower values for weight loss with ~10%. Furthermore, no significant differences in weight loss were observed among the coated fruits. This indicates the composite coating layers containing hydrophobic SF matrix deposited over the natural wax of fruit produced appropriate moisture retention, as previously noted with fruit active packaging applications based on other biopolymers such as chitosan [60,62,63] and pullulan [64]. Despite the possibility that the SF matrix might offer a barrier between the fruit and the external environment, the high content of *D*-limonene should be a crucial factor for the coating formulation presenting good preservation function at high humid and hot atmosphere. 

## 4. Conclusions

This study demonstrated that periodic mesoporous organosilica nanoparticles with ethylene (E-PMO) and phenylene (P-PMO) bridging moieties are a kind of effective carriers for encapsulating highly volatile fragrances. They had a *D*-limonene (Lim) uptake of 40–45 wt%, which was much higher than that of microporous nanomaterials reported previously. A distinct advantage of PMO NPs over traditional MSNs is that the former exhibited a steady and slower release pattern, associated with the hybrid nature of its channel walls. Under isothermal TGA conditions (30 °C, a N_2_ flow of 1 mL min^−1^), the time required to achieve a fragrance release at 50% for Lim@P-PMO (16.2 h) is 8.7 times longer than that of its MSN counterpart with close initial loading contents (~38 wt%). The release kinetics followed a Weibull model, and the release exponents indicated a Fickian diffusion mechanism. As a proof-of-concept application, the composite coating fabricated from regenerated silk fibroin and PMO NPs embedded with *D*-limonene was used to preserve perishable fruits. The coated mangoes can prolong their shelf life under humid and hot ambient conditions for up to 6 days. These results suggest that using PMO NPs as novel carrier materials has promising applications in active food packaging, cosmetics, home/personal care, and environmentally friendly pest control.

## Figures and Tables

**Table 1:** Synthetic conditions and pore parameters of siliceous NPs under study *^a^*.

Entry	Sample	*T*/°C; *t*/h	*d*_TEM_*^b^*/nm	*S*_BET_*^c^*/m^2^ g^−1^	*V*_p_*^d^*/cm^3^ g^−1^	*d*_p_*^e^*/nm	*d*_p,peak_*^f^*/nm
1	MSN	80; 2	81.9 ± 7.9	882.5	2.04	6.42	2.53
2	E-PMO	50; 6	104.4 ± 14.4	1082.3	1.45	3.71	2.82
3	E-PMO	80; 2	120.2 ± 19.8	904.3	0.73	2.58	2.53
4	P-PMO	50; 6	120.1 ± 22.6	1300.0	1.46	3.28	2.00
5	P-PMO	80; 2	105.4 ± 4.9	1654.0	1.34	2.52	2.26

*^a^* see the Section 2 and Supplementary Materials for the formulations in the material preparation. *^b^* Particle diameter determined using TEM observation. *^c^* Specific BET surface area. *^d^* BJH adsorption cumulative pore volume. *^e^* BJH average pore diameters calculated using the Barrett−Joyner−Halenda method. *^f^* The peaks seen on the BJH pore size distribution graph (Figure 1F).

**Table 2:** Release kinetic parameters of fragrances from different carriers in isothermal TGA conditions and the correlation coefficients to the mathematical models *^a^*.

Entry	Samples	Kinetic Model *^b^*	*R* ^2^	*k* (h^−1^)	*t*_50%_ (h)	*n*
1	Lim@MSN	first-order	0.9802	0.4981	1.86	–
2	Myr@MSN		0.9835	0.7125	1.26	–
3	Cym@MSN		0.9820	0.5631	1.61	–
4	Lim@E-PMO	Weibull	0.9900	0.4287	2.43	0.5895
5	Myr@E-PMO		0.9958	0.2930	2.23	0.4810
6	Cym@E-PMO		0.9947	0.1377	3.67	0.4624
7	Lim@P-PMO		0.9966	0.0203	16.20	0.3590
8	Myr@P-PMO		0.9991	0.0064	16.92	0.3970
9	Cym@P-PMO		0.9998	0.0309	17.56	0.6576

*^a^* The experiments were performed using a thermogravimetric analyzer at 30 °C and a N_2_ flow of 1.0 mL min^−1^. *k* and *t*_50%_ represent the release rate constant and the times required for the release of 50% of the fragrance, respectively. *^b^ R*^2^, the values of the correlation coefficient to the mathematical model; *n*, the exponent in the Weibull equation.

**Table 3:** Computed interaction energies of fragrance molecules with the bridging moieties in mesoporous silica and organosilica nanoparticles (kcal mol^−1^).

Fragrances	MSN	E-PMO	P-PMO
Lim	−11.99	−15.04	−19.98
Cym	−12.41	−13.84	−22.23
Myr	−9.91	−13.91	−21.04

## Data Availability

Not applicable to this article.

## Supplementary Materials

The following are available online at https://www.mdpi.com/article/10.3390/nano13010104/s1, Synthesis of mesoporous silica (MSN), Preparation of regenerated silk fibroin, Figure S1: NMR spectra of 1,4-bis(triethoxysilyl)benzene, Figure S2: Representative SEM images of NPs, Figure S3: TEM images of representative E-PMO NPs, Figure S4: TEM images of representative P-PMO NPs, Figure S5: DLS curves of PMO NPs, Figure S6: DTG curves of MSN, E-PMO, and P-PMO NPs, Figure S7: TGA curves of NPs before and after adsorption of myrcene/cymene, Figure S8: IR spectra of NPs before and after fragrance adsorption, Tables S1 and S2: Reaction conditions for PMO synthesis and characterization data. Reference [65] is cited in the supplementary materials.
