# Supplementary material for: Encapsulation of Volatile Monoterpene Fragrances in Mesoporous Organosilica Nanoparticles and Potential Application in Fruit Preservation
Source: Nanomaterials (Basel). 2022 Dec 25;13(1):104. doi: 10.3390/nano13010104 (PMC9823477; doi:10.3390/nano13010104)
Supplement: Supplementary file 1 [file nanomaterials-13-00104-s001.zip › nanomaterials-2104198-supplementary.pdf]

Encapsulation of Volatile Monoterpene Fragrances in Mesoporous Organosilica Nanoparticles and Potential Application in Fruit Preservation

Yuanjiang Zhao [1], Tianwen Bai [1], Yuhang Liu [1], Yichao Lv [1], Zhuxian Zhou [2], Youqing Shen [2] and Liming Jiang [1,*]

[1] MOE Key Laboratory of Macromolecular Synthesis and Functionalization, Department of Polymer Science and Engineering, Zhejiang University, Hangzhou 310027, China
[2] Key Laboratory of Biomass Chemical Engineering of Ministry of Education and Zhejiang Key Laboratory of Smart Biomaterials, College of Chemical and Biological Engineering, Zhejiang University, Hangzhou 310027, China
* Correspondence: cejlm@zju.edu.cn; Tel.: +86-571-87953727.

Index:

1. Synthesis of mesoporous silica (MSN)
2. Preparation of regenerated silk fibroin
3. Fig.S1. NMR spectra of 1,4-bis(triethoxysilyl)benzene
4. Fig.S2. Representative SEM images of NPs
5. Fig.S3. TEM images of representative E-PMO NPs
6. Fig.S4. TEM images of representative P-PMO NPs
7. Fig.S5. DLS curves of PMO NPs
8. Fig.S6. DTG curves of MSN, E-PMO, and P-PMO NPs
9. Fig.S7. IR spectra of NPs before and after fragrance adsorption
10. Fig.S8. TGA curves of NPs before and after adsorption of myrcene/cymene
11. Table S1 & S2. Reaction conditions for PMO synthesis and characterization data

S1. Synthesis of mesoporous silica nanoparticles (MSNs)

MSNs were synthesized by following the literature methods in [1]. A solution of cetyltrimethylammoniun bromide (CTAB, 1.0 g, 2.74 mmol) and aq. NaOH (3.5 mL, 2.0 mol/L) in 480 mL of deionized water was prepared, followed by heating the solution to 80 °C. Tetraethoxysilane (TEOS, 5 mL, 21.9 mmol) was added dropwise to the solution, and the mixture was allowed to stir for 2 h. The resulting white precipitate was filtered, washed with deionized water and ethanol alternately several times, and dried under a vacuum overnight. Finally, to remove the surfactant template, the solid product was calcined at 550 °C for 5 h in a muffle furnace, with a heating ramp of 2°/min.

S2. Preparation of regenerated silk fibroin

Regenerated silk fibroin (SF) was prepared according to previously reported methods with some modifications [2]. Briefly, Bombyx mori cocoons (procured in July 2021 from Tongxian Huicong Silk Textile Co. Ltd. Tongxiang, China) were boiled in an aqueous sodium carbonate solution (0.02 M) for 30 min and then rinsed thoroughly with deionized (DI) water. This process was repeated three times to completely remove the glue-like sericin and other impurities. The degummed silk was dissolved in an aqueous solution of LiBr (9.3 M) at 60 °C, and the resulting solution was dialyzed against DI water for 3 days using a dialysis membrane (molecular weight cutoff = 8−14 kDa; Yuanye Bio-Technology, Shanghai, China) and then lyophilized to obtain regenerated silk fibroin as a white sponge-like solid.

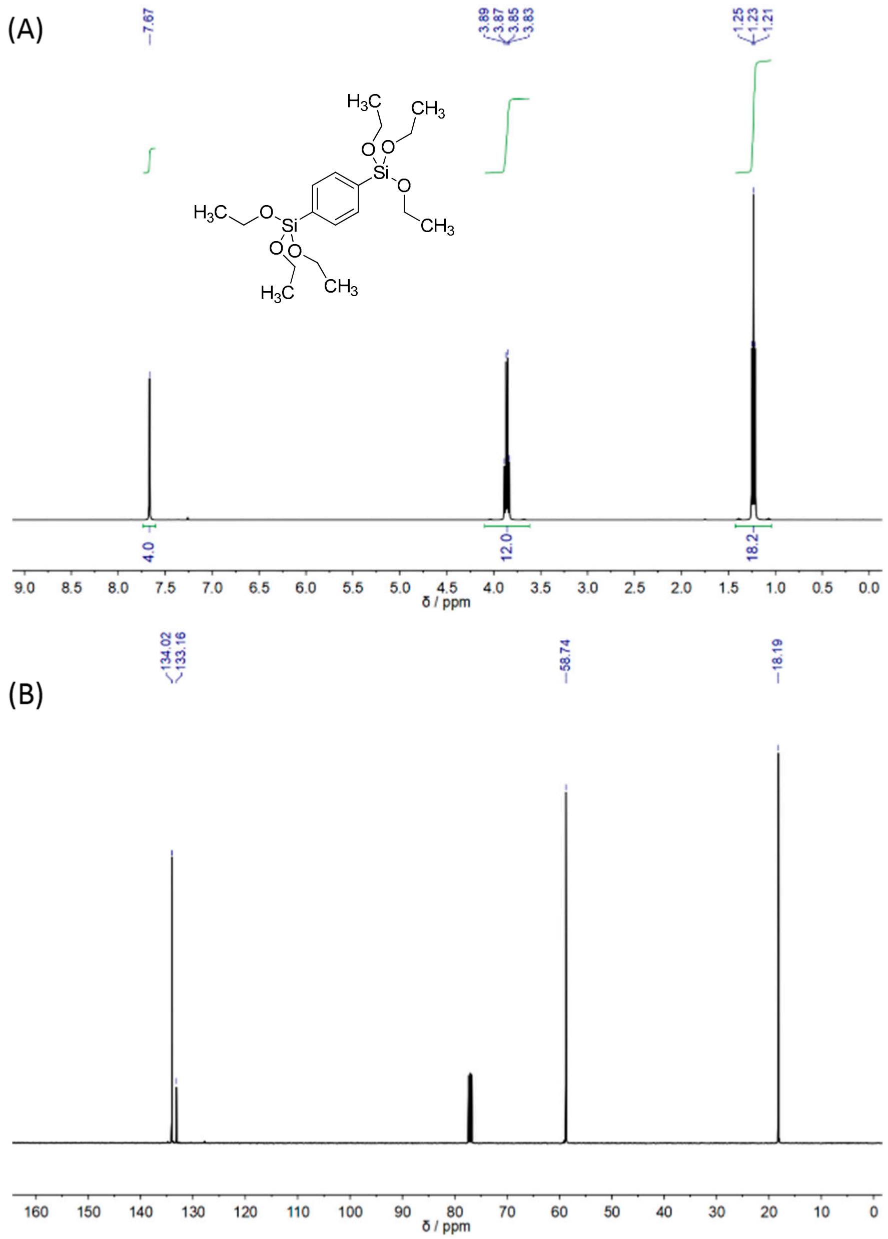

Figure S1. (A) ^{1}H NMR (400 MHz, $CDCl_3$) and (B) ^{13}C NMR (101 MHz, $CDCl_3$) spectra of 1,4-di(triethoxysilyl)benzene.

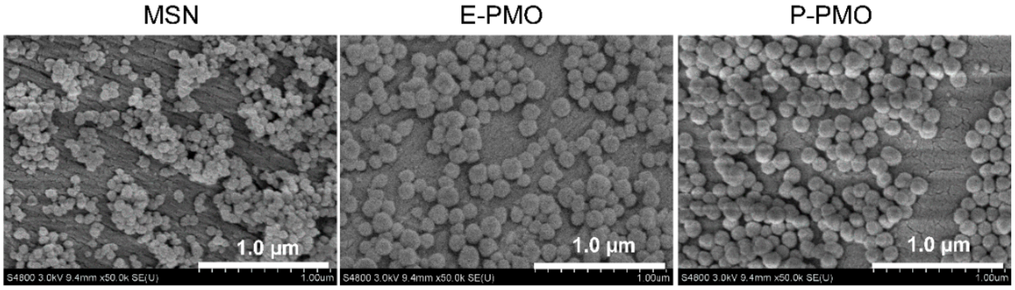

Figure S2. Representative SEM micrographs of MSN, E-PMO (No.2 in Table S1), and P-PMO nanoparticles (No.4 in Table S2).

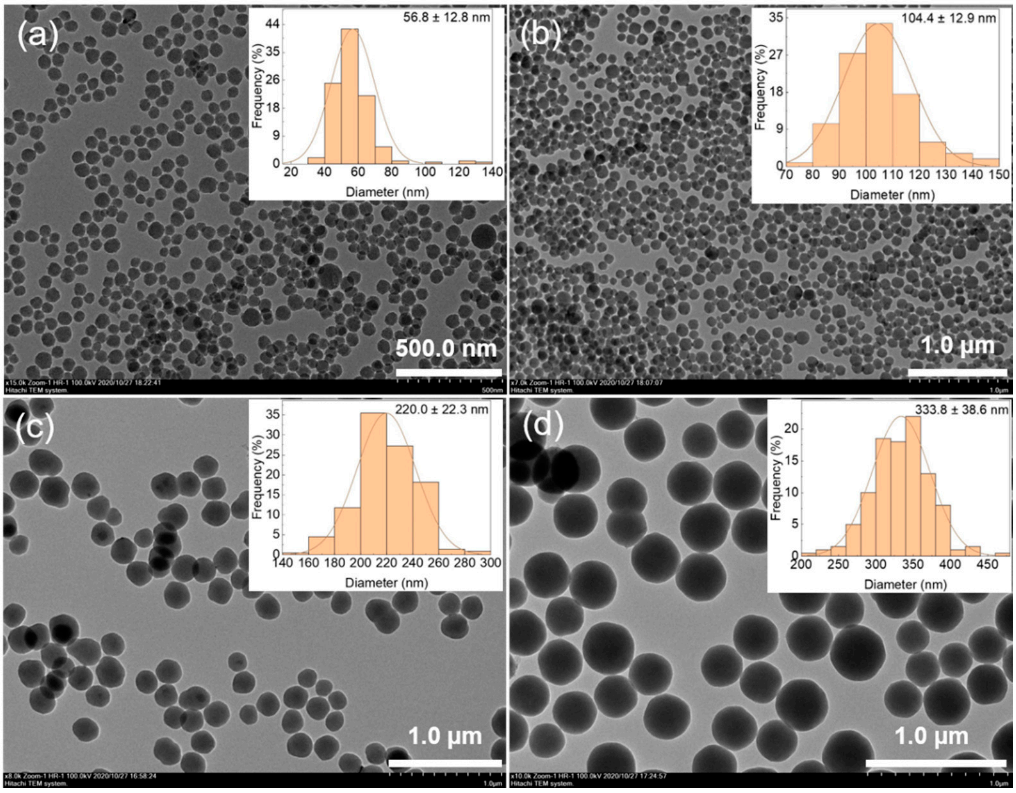

Figure S3. TEM images of representative E-PMO samples and the corresponding histograms of the distribution of particle diameters.

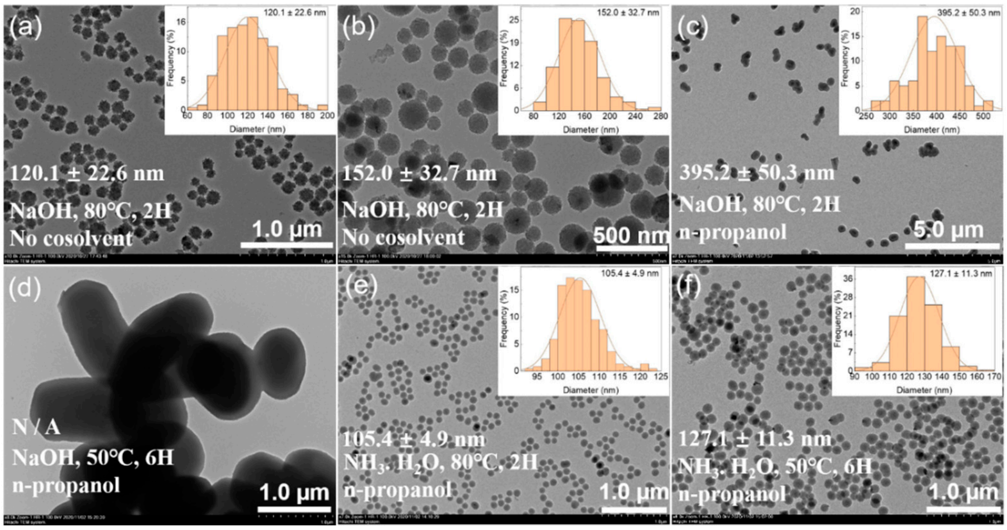

Figure S4. TEM images of representative P-PMO samples and the corresponding histograms of the distribution of particle diameters.

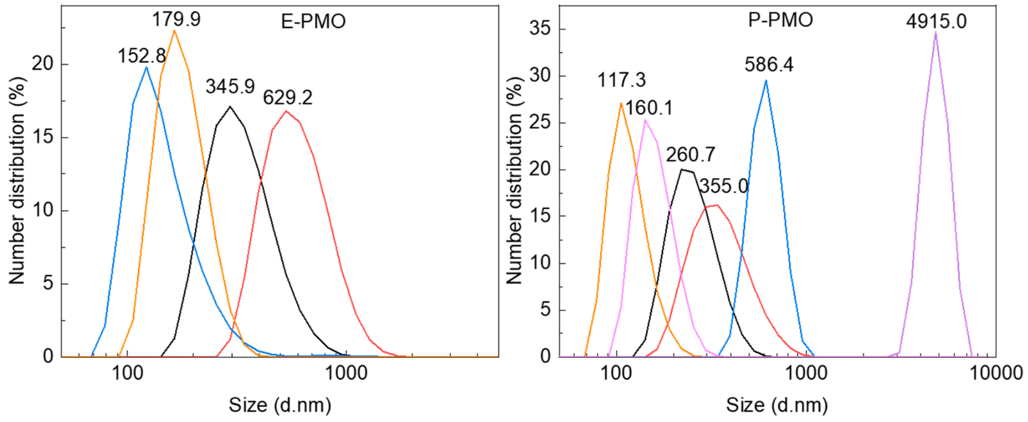

Figure S5. Hydrodynamic diameters of PMO NPs as determined using DLS measurements (see Tables S1 and S2).

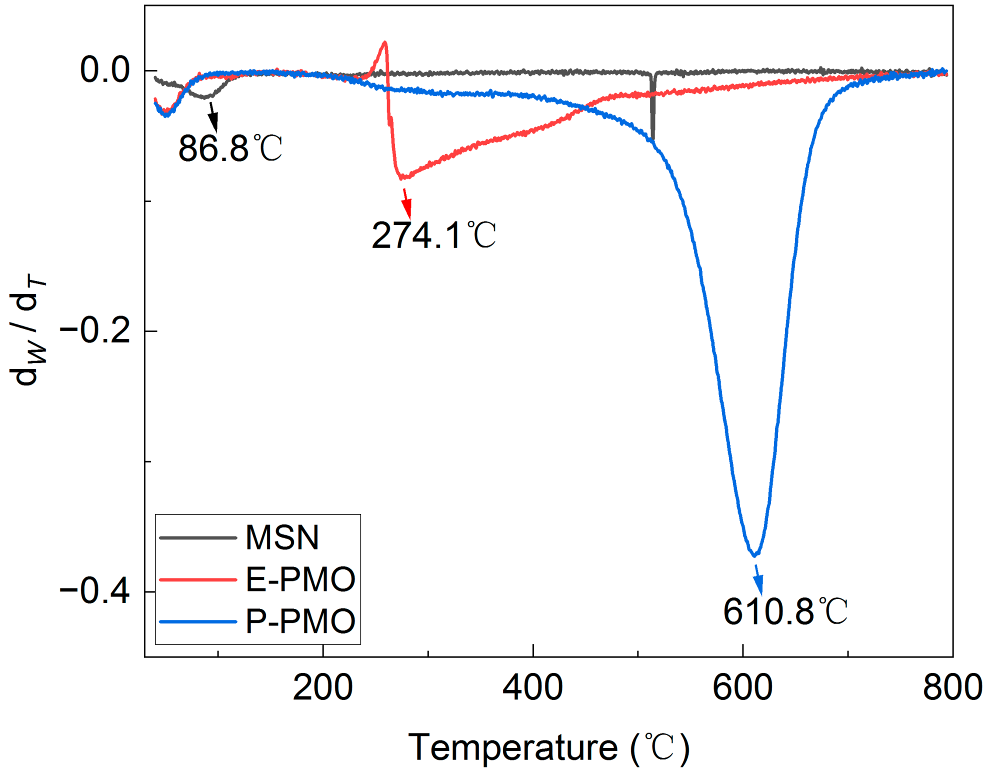

Figure S6. DTG curves of the carrier materials (MSN, E-PMO, and P-PMO).

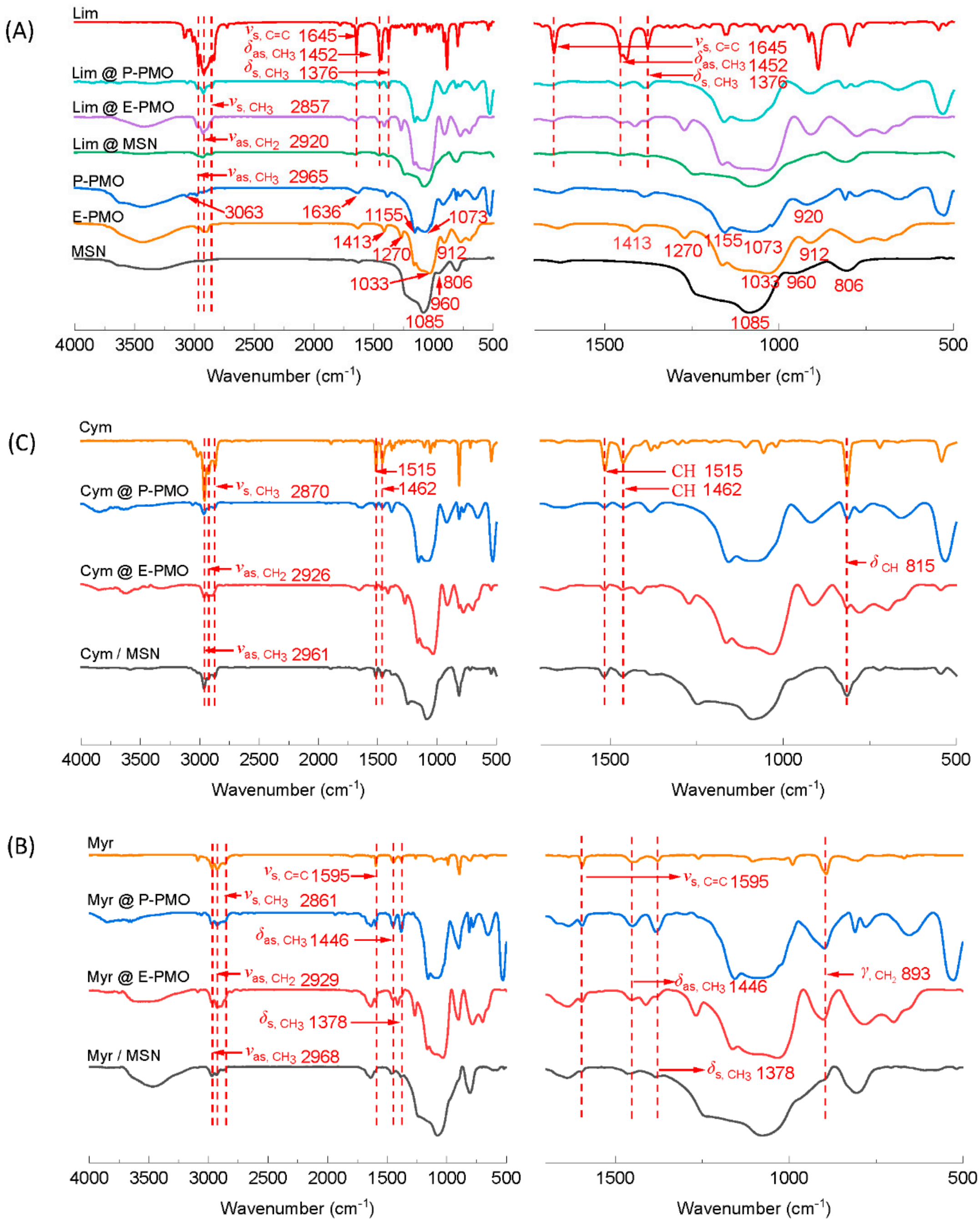

Figure S7. FT-IR spectra of pure fragrances (A) *D*-limonene, (B) myrcene, (C) cymene, and the corresponding nanocomposites.

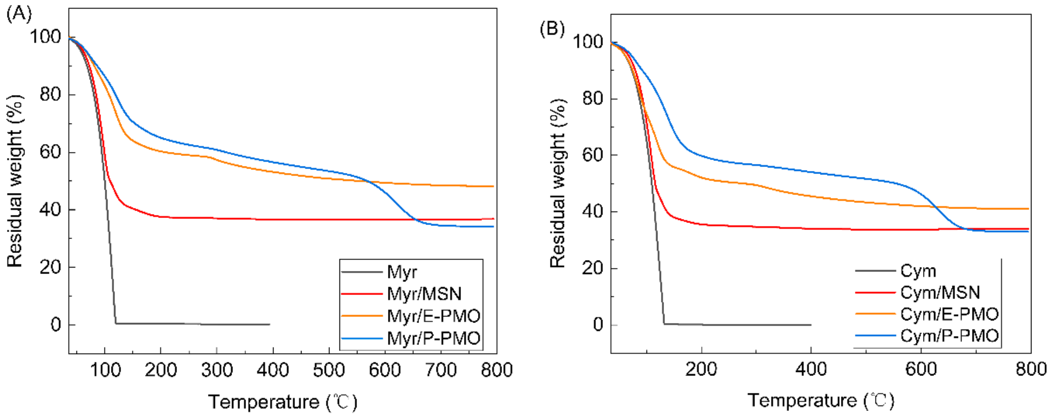

Figure S8. TGA curves of pure fragrances and the corresponding nanocomposites.

Table S1. Reaction conditions for E-PMO synthesis and partial characterization data

No.	Reactant composition [a]			Reaction temp. (°C) / time (h)	Particle characterization		
	NH_4OH (mL)	NaOH (mL)	BTE (mmol)		D_{TEM} (nm)	D_{DLS} (nm)	ζ-potential (mV)
1	1.3	–	0.92	50 / 6	56.8 ± 12.8	152.8 ± 6.0	–24.8 ± 0.2
2	2.6	–	0.92	50 / 6	104.4 ± 12.9	179.9 ± 0.1	–22.1 ± 0.1
3	–	0.6	1.12	80 / 2	220.0 ± 22.3	345.9 ± 11.8	–18.4 ± 0.3
4	–	0.6	2.24	80 / 2	333.8 ± 38.6	629.2 ± 35.0	–12.8 ± 0.4

[a] Solvent: water 96 mL; CTAB 0.21 g, 27 wt.% NH_4OH, 2.0 M NaOH (aq.), BTE = 1,2-bis(triethoxysilyl)ethane.

Table S2. Reaction conditions for P-PMO synthesis and characterization results

No.	Reactant composition [a]			Reaction temp. (°C) / time (h)	Particle characterization		
	NH_4OH (mL)	NaOH (mL)	BTB (mmol)		D_{TEM} (nm)	D_{DLS} (nm)	ζ-potential (mV)
1[b]	–	0.6	0.76	80 / 2	120.1 ± 22.6	260.7 ± 2.6	–22.6 ± 0.4
2[b]	–	0.6	1.26	80 / 2	152.0 ± 32.7	355.0 ± 11.8	–23.7 ± 1.0
3	–	0.6	0.76	80 / 2	395.2 ± 50.3	586.4 ± 44.7	–17.2 ± 0.3
4	2.0	–	0.76	80 / 2	105.4 ± 4.9	117.3 ± 1.1	–24.7 ± 1.0
5[b]	–	0.6	0.76	50 / 6	N/A	4915.0±103.9	–15.1 ± 0.2
6	2.0	–	0.76	50 / 6	127.1 ± 11.3	160.1 ± 3.1	–23.9 ± 0.6

[a] Solvent: water (86 ml) + *n*-propanol (10 ml); CTAB 0.21 g, 27 wt.% aq. NH_4OH, 2.0 M NaOH (aq.), BTB = 1,4-bis(triethoxysilyl)benzene. [b] in the absence of co-solvent *n*-propanol.

References:

[1] Knežević, N.Ž.; Ilić, N.; Đokić, V.; Petrović, R.; Janaćković, Đ. Mesoporous silica and organosilica nanomaterials as UV-blocking agents, ACS Appl. Mater. Interfaces 2018, 10, 20231.

[2] Kim, U. J.; Kaplan, D. L. Three-dimensional aqueous-derived biomaterial scaffolds from silk fibroin. Biomaterials 2005, 26, 2775.
